# Primary Human Microglia Are Phagocytically Active and Respond to *Borrelia burgdorferi* With Upregulation of Chemokines and Cytokines

**DOI:** 10.3389/fmicb.2018.00811

**Published:** 2018-04-25

**Authors:** Jacob R. Greenmyer, Robert A. Gaultney, Catherine A. Brissette, John A. Watt

**Affiliations:** ^1^Department of Biomedical Sciences, School of Medicine and Health Sciences, University of North Dakota, Grand Forks, ND, United States; ^2^Institut Pasteur, Paris, France

**Keywords:** *Borrelia burgdorferi*, microglia, neuroborreliosis, Lyme disease, phagocytosis

## Abstract

The Lyme disease causing bacterium *Borrelia burgdorferi* has an affinity for the central nervous system (CNS) and has been isolated from human cerebral spinal fluid by 18 days following *Ixodes scapularis* tick bite. Signaling from resident immune cells of the CNS could enhance CNS penetration by *B. burgdorferi* and activated immune cells through the blood brain barrier resulting in multiple neurological complications, collectively termed neuroborreliosis. The ensuing symptoms of neurological impairment likely arise from a glial-driven, host inflammatory response to *B. burgdorferi*. To date, however, the mechanism by which the bacterium initiates neuroinflammation leading to neural dysfunction remains unclear. We hypothesized that dead *B. burgdorferi* and bacterial debris persist in the CNS in spite of antibiotic treatment and contribute to the continuing inflammatory response in the CNS. To test our hypothesis, cultures of primary human microglia were incubated with live, antibiotic-killed and antibiotic-killed sonicated *B. burgdorferi* to define the response of microglia to different forms of the bacterium. We demonstrate that primary human microglia treated with *B. burgdorferi* show increased expression of pattern recognition receptors and genes known to be involved with cytoskeletal rearrangement and phagocytosis including MARCO, SCARB1, PLA2, PLD2, CD14, and TLR3. In addition, we observed increased expression and secretion of pro-inflammatory mediators and neurotrophic factors such as IL-6, IL-8, CXCL-1, and CXCL-10. Our data also indicate that *B. burgdorferi* interacts with the cell surface of primary human microglia and may be internalized following this initial interaction. Furthermore, our results indicate that dead and sonicated forms of *B. burgdorferi* induce a significantly larger inflammatory response than live bacteria. Our results support our hypothesis and provide evidence that microglia contribute to the damaging inflammatory events associated with neuroborreliosis.

## Introduction

The bacterium *B. burgdorferi* causes Lyme disease, which affects many tissues and organs, including the CNS. Lyme neuroborreliosis may manifest as meningitis, cranial neuritis, facial nerve palsy, encephalitis, or peripheral nerve disease (Rupprecht et al., 2008; Ramesh et al., 2013; Halperin, 2015). Additional rare complications can include CNS vasculitis and hemorrhagic stroke (Fallon et al., 2010; Back et al., 2013). While most patients respond well to antibiotics, some experience long-term sequelae including depressive states, decreases in verbal fluency, fatigue, sleep disruptions, emotional lability, and short-term memory problems (Fallon and Nields, 1994; Gustaw et al., 2001; Fallon et al., 2008, 2010; Ljostad and Mygland, 2010). Indeed, approximately 20% of patients treated for Lyme disease experience PTLDS. However, the cause of persistent, lingering neurological symptoms in patients following antibiotic treatment is unknown.

Examination of tissues from patients with neuroborreliosis is limited, but there is evidence for inflammatory changes in the brains of patients, as well as from rhesus macaque models (Sumiya et al., 1997; Ramesh et al., 2013, 2015). Indeed, post-mortem pathological analysis from patients with clinically diagnosed neuroborreliosis revealed lymphocytic infiltrates in the CNS along with widespread microglial activation (Bertrand et al., 1999). While the cause of neurologic symptoms following antibiotic treatment is unclear, dead spirochetes and/or their debris can provide a constant immune stimulus that contributes to inflammatory pathogenic processes (Bockenstedt et al., 2012; Embers et al., 2012, 2017; Wormser et al., 2012; Parthasarathy et al., 2013). Dead spirochetes and spirochetal antigens have been found to persist within injured tissues in human patients with Lyme disease, and spirochetal antigens persist in the joints of mice following antibiotic treatment (Steere et al., 1988; Nanagara et al., 1996; Bockenstedt et al., 2012). Whether such material persists in the CNS of patients is unknown; however, *in vitro* studies demonstrate that non-viable *B. burgdorferi* can stimulate the secretion of inflammatory mediators from human CNS cells such as oligodendrocytes (Parthasarathy et al., 2013). Collectively, these reports indicate that the presence of spirochetal debris following antibiotic treatment may drive persistent inflammation.

Microglia are resident CNS cells that respond to injury. These dynamic cells of myeloid origin possess immune receptors such as Fc receptors and TLRs, and as such are poised to infections with microbes such as *B. burgdorferi* (Lee et al., 2013). Indeed, upon stimulation with *B. burgdorferi* lysate, expression of TLR1 and TLR2 increases leading to the activation of TLR-linked intracellular signaling pathways (Cassiani-Ingoni et al., 2006; Parthasarathy and Philipp, 2015). Microglia are also facultative phagocytes and, in addition to secreting pro-inflammatory cytokines, produce neurotropic and neuroprotective factors. Hence, microglia are recognized as innate immune cells of the CNS but also have roles in brain homeostasis and tissue repair (Blaylock, 2013). Inflammatory and homeostatic responses must be balanced to eliminate pathogens and repair wounds while preserving brain tissue and minimizing neurological impairment. Continuous stimulation of microglia drives a pro-inflammatory profile in the CNS and disrupt homeostasis.

We hypothesized that dead *B. burgdorferi* and debris persist in the CNS following antibiotic treatment and promote a chronic inflammatory response in the CNS through interaction with microglial cells. In this study, we compared the innate immune and phagocytic responses of highly pure cultures of primary human microglia to live or killed *B. burgdorferi*. We demonstrate that primary human microglia treated with *B. burgdorferi* increase expression of genes involved with innate immunity, cytoskeletal rearrangement, and phagocytosis, and secrete pro-inflammatory mediators and neurotrophic factors. We also visualized *B. burgdorferi*–microglia interactions and demonstrate that the spirochete adheres to the cell surface and may be internalized.

## Materials and Methods

### Microglial Culture

**FIGURE 1:**
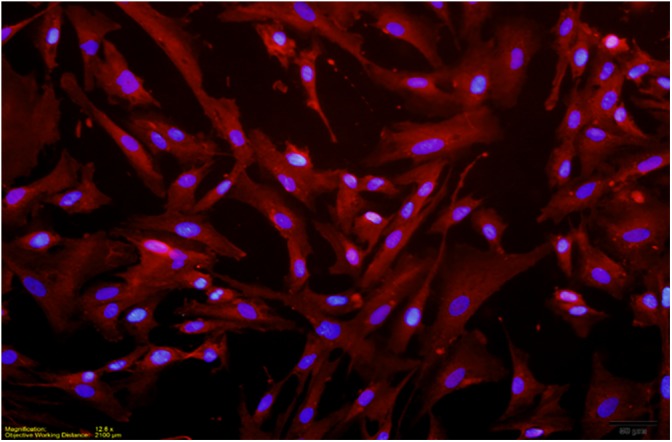
Dual localization of DAPI stained nuclei and Texas Red labeled IBA-1 (red) in passage 3 culture. Two independent immunocytochemistry experiments were performed, with two replicate coverslips per experiment. One count was made per quadrant of coverslip to give a total of eight counts for each independent experiment. Cell purity in a passage 3 culture was revealed to be >95%.

Primary human microglial cells were purchased from ScienCell Research Laboratories (Carlsbad, CA, United States; catalog #1901). Microglial cells were maintained on T75 flasks coated with poly-L-lysine (Corning, 2 μg/cm^2^, T-75) in microglia medium (ScienCell, catalog # 1901) supplemented with 100 units/mL of penicillin and 100 μg/mL of streptomycin. In addition, cultures contained microglia growth supplement (MGS, catalog # 1952). Prior to *B. burgdorferi* stimulation, medium was replaced with antibiotic-free medium. Microglia were used at passages 2 or 3 at >85% confluence. *B. burgdorferi* at a MOI of 10:1 bacteria:cells was used to stimulate microglia for 24 or 72 h. Cell purity was determined at all passages using immunocytochemical labeling of parallel cultures grown on 12 mm glass coverslips (Deutsch Deckglaser-NeuVita Inc., Germany) coated with poly-L-lysine (MilliporeSigma, St. Louis, MO, United States) with a specific marker for microglia rabbit anti IBA-1 (Wako Chemicals, Richmond, VA, United States). 97% of cells were Iba-1 positive at passage 2 as were 95% of cells at passage 3, indicating high levels of culture purity as determined by counting the number of IBA-1 expressing cells vs. DAPI stained nuclei (**Figure 1**). Experimental protocols and commercially available primary cell cultures utilized in these studies followed the University of North Dakota IRB guidelines as stated in section 2.19 “Commercially Available Human Biological Specimens (45 CFR 46.102, 46.103, and 46.116)” and do not require IRB review.

### *Borrelia burgdorferi* Culture

Virulent *B. burgdorferi* strain B31 MI-16 (Fraser et al., 1997; Casjens et al., 2000; Miller et al., 2003), avirulent derivative e2 (Babb et al., 2004), or *gfp*-expressing *B. burgdorferi* (kind gift of M.A. Motaleb, Eastern Carolina University; Sultan et al., 2015) were grown at 34°C to cell densities of approximately 1 × 10^8^/mL in modified Barbour-Stoenner-Kelly II (BSK-II) medium (Zückert, 2007). For stimulation of microglial cultures, *B. burgdorferi* were centrifuged at 6,000 ×*g*, washed 3X with PBS, and resuspended in microglia medium containing no antibiotics. Bacterial counts were determined using a Petroff-Hausser chamber and viewed using dark field microscopy. For bacterial killing, 1 × 10^8^ *B. burgdorferi*/mL were treated with ceftriaxone (MilliporeSigma) for 72 h at 5X the minimum inhibitory concentration (Veinovic et al., 2013). Ceftriaxone was chosen as the antibiotic as it is the preferred treatment for neurological symptoms according to both United States and European guidelines for human treatment of Lyme disease (Mygland et al., 2010; Hu, 2012). Complete killing of *B. burgdorferi* was confirmed by darkfield microscopy and subculture into fresh BSKII medium (data not shown). For stimulation experiments performed with *B. burgdorferi* sonicate, cultures were subjected to five pulses (amplitude 4 for 12–15 s each) with a probe sonicator (Fisher Scientific, Pittsburgh, PA, United States; Model 120). A 10:1 MOI was used to stimulate microglia cells with live *B. burgdorferi*. An equivalent of 10:1 MOI was used to stimulate cell cultures with antibiotic-killed *B. burgdorferi*. The amount of sonicate that was used to stimulate cultures of microglia was equivalent to 10:1 MOI and was determined by the concentration of *B. burgdorferi* in media prior to spirochetal disruption. In this manner, stimulation experiments were performed at a 10:1 MOI of live or dead bacteria, or the amount of sonicate that would have resulted in a 10:1 MOI had it not been sonicated.

### Nucleic Acid Isolation and Synthesis

Total RNA was isolated via the RNeasy kit (Qiagen, Valencia, CA, United States). After aspiration of media from stimulated cultures, adherent cells were washed 3X with warm, sterile PBS. Cells were suspended with trypsin-EDTA (ScienCell) and all enzymatic activity neutralized with the addition of microglial medium containing 5% fetal bovine serum. Buffer RLT (Qiagen) was used to lyse cells. Genomic DNA was then removed by on-column DNA digestion with RNase-Free DNase Set (catalog # 79254, Qiagen). RNA was then concentrated using a the Qiagen RNeasy MinElute Cleanup Kit (catalog # 74204). RNA quality and concentration was assessed by spectrometry (Epoch, Bio-Tek, Winooski, VT, United States). cDNA was synthesized with Qiagen RT^2^ First Strand Kit (Catalog # 330401) following manufacturer’s instructions.

### Gene Expression Analysis by Quantitative Real-Time RT-PCR (qPCR)

The expression of chemokine, cytokine, and neurotrophin genes, as well as those associated with phagocytosis, was determined first with RT^2^ Profiler PCR arrays (Qiagen) on a Bio-Rad myIQ2 Real-Time PCR instrument. The manufacturer’s software was used to analyze the data. Alterations in individual genes were confirmed using individual PCR primer sets for qRT-PCR (Qiagen; **Table 1**). Individual reactions contained 5.5 μl nuclease-free water, 2 μl primer mix (both forward and reverse) at 10 μM, and 12.5 μl Bio-Rad Sybr Green Supermix ±5 μl template DNA or no template control (nuclease-free water). Forty cycles of PCTR were performed following an initial 10 min denaturation at 95°C. For each cycle, a 1 min annealing step at 60°C preceded a 15-s melting interval at 95°C. Melting curves were obtained using a stepped temperature gradient of 0.5°C × 10 s starting at 60°C. Transcript expression levels were then compared to standard housekeeping genes (β-actin and GAPDH) and to those of untreated cells using the 2^-ΔΔCT^ method, where C_T_ (threshold cycle). This method was used on each individual example with the untreated sample as the comparator (Schmittgen and Livak, 2008). Triplicate samples were analyzed from a minimum of three independent biological replicates for each time point.

**Table 1:** Primer sets used for quantitative RT-PCR.

Primer name	RefSeq accession	Qiagen catalog #
B-actin	NM_001101	PPH00073G
GAPDH	NM_002046	PPH00150F
IL-8	NM_000584	PPH00568A
CXCL-1	NM_001511	PPH00696C
CXCL-10	NM_001565	PPH00765E
VEGF	NM_003376	PPH00251C
LIF	NM_002309	PPH00813F
IL-6	NM_000600	PPH00560C
IL-15	NM_000585	PPH00694B
CD14	NM_000591	PPH05723A
MARCO	NM_006770	PPH09783A
CSF1	NM_000757	PPH00124B
C3	NM_000064	PPH01185E
FCER1G	NM_004106	PPH02628B
CD36	NM_000072	PPH01356A
TLR3	NM_003265	PPH01803E
WNT5A	NM_003392	PPH02410A
COLEC12	NM_130386	PPH08828C

### Enzyme-Linked Immunosorbent Assay (ELISA)

Following microglial stimulation, culture supernatants were collected, aliquoted for single use and stored at -80°C. ELISA kits were purchased from R&D Systems (Minneapolis, MN, United States) and were used following manufacturer’s instructions. Briefly, plates were coated overnight with 100 μl of specific capture antibody (e.g., anti-IL-8) followed by multiple wash cycles. Sample standards and controls (100 μl) were then added to individual wells and incubated for 2 h at room temperature. Plates were then washed 3X and 100 μl of antibody conjugate was added to each well, and incubated for 2-h at room temperature and finally chromogenic detection of desired host cell factor. Plates reads were performed at an optical density of 450 nm on a BioTek Epoch plate reader. Triplicate samples from each treatment group were collected and pooled. Data represent the means and standard errors from at least two independent biological replicates analyzed in triplicate per time point.

### Immunocytochemistry

Microglia were maintained on poly-L-lysine coated glass coverslips (Neurovita, Germany) in microglia medium (seeding density of 20,000 cells/coverslip). Microglia were stimulated by exposure to *B. burgdorferi* MOI 10:1 for 24 or 72 h. Microglia were fixed for 15 min in buffered 4% paraformaldehyde and washed 3X with PBS. The cells were given three 10 min PBS plus 0.1% Triton x100 (PBS-T) washes and were incubated for 1 h at room temperature in blocking serum. Blocking serum consisted of 4% normal serum of the species of the secondary antibody. The cells were probed with rabbit anti-IBA-1; 1:500 dilution (Wako Chemicals) diluted in blocking serum and incubated overnight at 4°C. Cells were washed 3X with PBS-T and incubated for 1 h in a solution of biotinylated goat anti-rabbit IgG, (1:500 dilution; Jackson Immunoresearch, West Grove, PA, United States) diluted in blocking serum. Following three more washes with PBS-T, the cells were incubated in a solution of streptavidin-AlexaFluor 594 for 1 h (1:1000 dilution; Thermo Fisher, Grand Island, NY, United States; cat #S11227). Cells were then washed in PBS 3X and coverslipped with Vectashield containing DAPI (Vector Laboratories, Burlingame, CA, United States). Immunostained coverslips were visualized with fluorescent microscopy using an Olympus BX-51 fluorescence microscope with attached DP-71 digital camera and dedicated CellSens Standard software (Olympus, Waltham, MA, United States).

### Confocal Microscopy

Coverslips were coated with collagen at 5 μg/cm^2^ using a ScienCell Collagen I-Cell Culture Surface Coating Kit (Cat #8188) according to manufacturer’s instructions. Microglia were maintained on collagen-coated glass coverslips in microglia medium (seeding density of 20,000 cells/coverslip). Microglia were stimulated by exposure to *B. burgdorferi* MOI 10:1 for 24 h, or with 5 × 10^6^ beads per well (∼100 ± 25 beads per cell) of green Fluoresbrite Plain YG 1.0 micron microspheres with excitation maxima and emission maxima of 441 nm and 485 nm, respectively (Polysciences Inc., Warrington, PA, United States; Cat# 17154). Cells were fixed for 15 min in buffered 4% paraformaldehyde and washed three times with PBS. The cells were blocked in PBS buffer until labeled with IBA-1 as described above. Images were acquired on an Olympus FV300 confocal microscope. Z stacks were taken at 0.225 μm/image with a Kallman of 3. Images were viewed and edited with Imaris software.

### Statistical Analysis

Enzyme-linked immunosorbent assay experiments were carried out two times (24-h samples) or three times (72-h samples and control samples) in biologically independent experiments with triplicate replicates. Chemokine and cytokine qPCR experiments were carried out three or four times in independent experiments with triplicate replicates. Phagocytosis experiments were carried out between four and seven times in biologically independent experiments with triplicate replicates. Statistical significance for ELISAs was determined by running a one-way ANOVA with a *post hoc* Student–Newman–Keuls (SNK) test. Statistical significance for qPCR experiments was determined by running a one-way ANOVA with a *post hoc* Dunn’s test.

## Results

### Characterization of Microglia Culture Purity

To verify the purity of our primary human microglial cultures, cultures of passage 2–3 microglia were incubated with anti-IBA-1 antibody and counterstained with DAPI. Culture purity was determined by dividing the number of cells immunoreactive for IBA-1 staining (microglia) with the total number of nuclei (DAPI). At passage 2, primary human microglial cultures were >97% pure; at passage 3, primary human microglial cultures were >95% pure (**Figure 1**). No changes in morphological characteristics were observed through the passages.

### Differential Chemokine Gene Expression in Human Microglia Cells Stimulated With *B. burgdorferi*

Primary cultures of human microglia cells were stimulated with virulent, non-virulent, and antibiotic killed *B. burgdorferi* for 24–72 h. RNA was extracted from stimulated and control cells, cDNA synthesized for real-time PCR array analysis. There was very little variation between technical replicates across the array, with standard deviations averaging less than 1 threshold cycle (data not shown). Several genes were differentially upregulated more than threefold by non-virulent *B. burgdorferi*, virulent *B. burgdorferi*, and antibiotic-killed bacteria, including neutrophil chemoattractants (IL-8), growth/differentiation factors (CSF-1, GDF-5, LIF), and cytokines IL-6, IL-15, and VEGF (**Table 2**).

**Table 2:** Transcriptional profiling of chemokine gene expression in microglial cells upon stimulation with *B. burgdorferi.*

Gene	Non-virulent	Live	Abx	Function
BMP1	105.4	1209.3	760.0	Bone morphogenic protein; metalloprotease
CSF1	35.5	276.2	194.0	Macrophage colony stimulating factor
GDF5	74.0	235.5	205.0	Growth differentiation factor
IL15	112.2	116.9	95.6	IL2-like cytokine affecting T cell differentiation
IL6	190.0	519.1	661.6	Cytokine important for acute phase response
IL8	340.1	398.9	349.7	Neutrophil chemoattractant
LIF	233.9	648.0	675.5	Leukemia inhibitory factor
VEGFA	182.2	544.9	604.6	Angiogenic factor

*Values shown correspond to the mean ratio of triplicate measurements between normalized gene intensity values determined after 72 h of stimulation with 10:1 B. burgdorferi strain B31 (Live), non-virulent B. burgdorferi B31-e2 (NV), or antibiotic-killed B. burgdorferi (Dead) compared with gene intensity values from unstimulated cells*.

**FIGURE 2:**
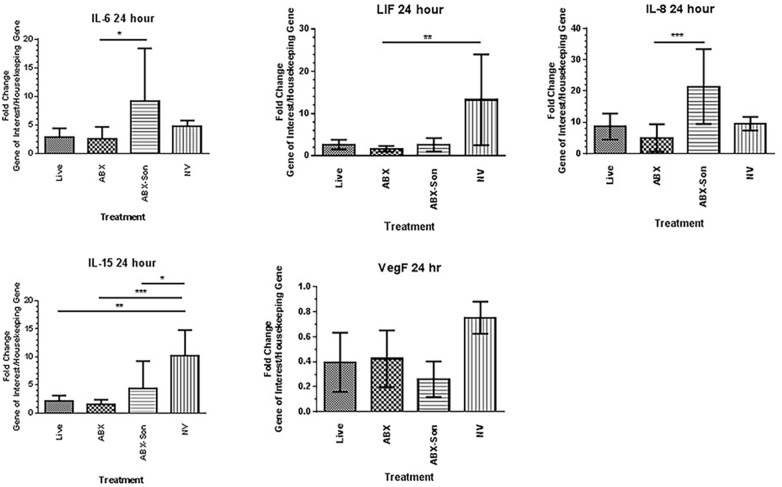
Validation of differentially regulated genes by qPCR array analysis. Primary human microglia were stimulated with live virulent (V), non-virulent (NV), antibiotic-killed (Abx), or antibiotic-treated and sonicated (Abx/Son) *B. burgdorferi* for 24 or 72 h. Data are representative of two biological replicates per time point, with each PCR reaction run in triplicate. Data were normalized to transcription levels of two independent housekeeping genes and are expressed as fold change versus no spirochete control. Error bars represent SEM. Statistical significance for qPCR experiments was determined by running a one-way ANOVA with a *post hoc* Dunn’s test (^∗^*p* < 0.05, ^∗∗^*p* < 0.01, ^∗∗∗^*p* < 0.001).

Transcription of selected genes from the PCR array were confirmed at 24 and 72 h after stimulation with live virulent, non-virulent, antibiotic-killed, and antibiotic-killed sonicated *B. burgdorferi*. A similar trend was observed for several transcripts at 24 h, including IL-6, IL-8, IL-15, LIF, and VEGF (**Figure 2**).

**FIGURE 3:**
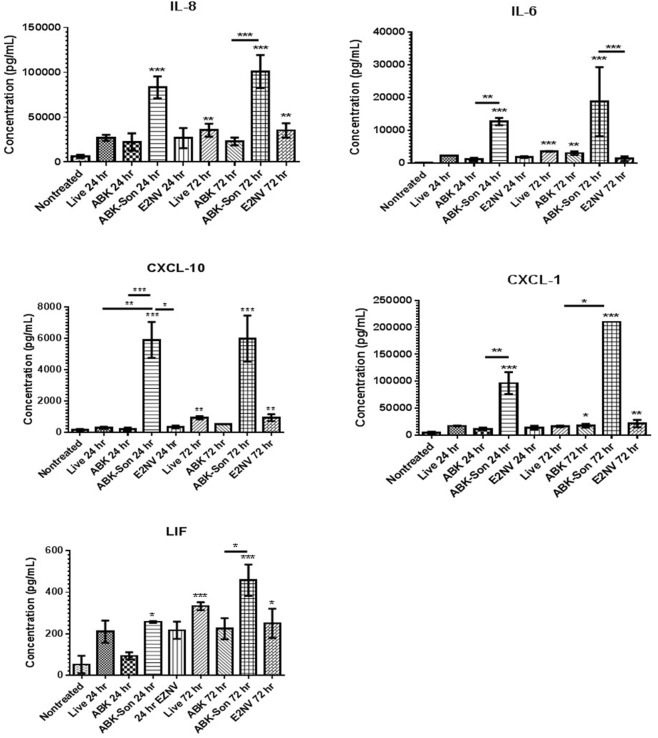
*B. burgdorferi*-stimulated microglia protein expression. Primary human microglia were stimulated with live virulent, non-virulent, antibiotic-killed or antibiotic-treated and sonicated *B. burgdorferi* for 24 or 72 h as described in “Materials and Methods.” Cytokine and chemokine concentrations were measured using ELISA analysis (R&D Systems). Data represent two biological replicates per time point, with each sample run in triplicate. Error bars represent SD. Statistical significance for ELISAs was determined by running a one-way ANOVA with a *post hoc* Student–Newman–Keuls (SNK) test (^∗^*p* < 0.05, ^∗∗^*p* < 0.01, ^∗∗∗^*p* < 0.001).

To determine if these transcriptional changes were biologically relevant, we measured the production of cytokines, chemokines, and neurotrophins secreted from microglia stimulated with live virulent, non-virulent, and antibiotic-killed and antibiotic-killed sonicated spirochetes for 24–72 h. We quantified supernatant protein concentrations of IL-6, IL-8, LIF, CXCL-1, and CXCL-10 by ELISA. Our results demonstrate elevated protein levels for all six proteins examined when compared to non-treatment controls with the highest concentrations of protein associated with the antibiotic-treated and sonicated *B. burgdorferi.* Chemokines IL-8, CXCL-1, and CXCL-10 and the cytokine IL-6 were generally secreted in a temporal pattern, with highest secretion occurring 72 h after cell stimulation when compared to the 24 h time point (**Figure 3**).

### Differential Phagocytosis-Related Gene Expression in Human Microglia Cells in Response to *B. burgdorferi*

Primary cultures of human microglia cells were stimulated with exponential phase live, antibiotic-killed and antibiotic-killed sonicated spirochetes for 24 h. Again, we utilized commercial qPCR arrays to quantify the transcription of a panel of human phagocytosis-related genes. Surprisingly, no genes were upregulated when stimulated with live *B. burgdorferi* (data not shown). Because antibiotic-killed *B. burgdorferi* had elicited robust chemokine expression, we investigated whether primary human microglia would respond to dead *B. burgdorferi*. Bacteria were either killed with antibiotics or killed with antibiotics and disrupted by sonication. In contrast to live *B. burgdorferi*, several genes were upregulated more than threefold by dead *B. burgdorferi*, including scavenger receptors (MARCO, SCARB1), PLAs (PLA2, PLD2), and pattern recognition receptors (CD14, TLR3) (**Table 3**).

**Table 3:** Transcriptional profiling of phagocytosis-related gene expression in microglial cells in response to *B. burgdorferi.*

Gene	Abk	Abks	Function
CD14	10.3	17.4	Co-receptor for PAMPS
CSF1	2.4	8.8	Macrophage colony stimulating factor
LYN	3.7	2.8	Src family tyrosine kinase
MARCO	8.3	9.2	Scavenger receptor
PLA2G4	3.1	5.9	Phospholipase A2
PLD2	3.6	4.3	Phospholipase D2
SCARB1	3.8	5.8	Scavenger receptor
TLR3	3.4	4.7	Toll-like receptor 3; endosomal TLR
WNT5A	13.4	6.8	Wingless homolog; stimulates phagocytosis

*Values represent the mean ratio of triplicate measurements determined between normalized gene intensity values after 24 h of stimulation with 10:1 antibiotic-killed B. burgdorferi strain B31 or antibiotic-killed and sonicated B. burgdorferi (ABKS) compared with gene intensity values from unstimulated cells*.

**FIGURE 4:**
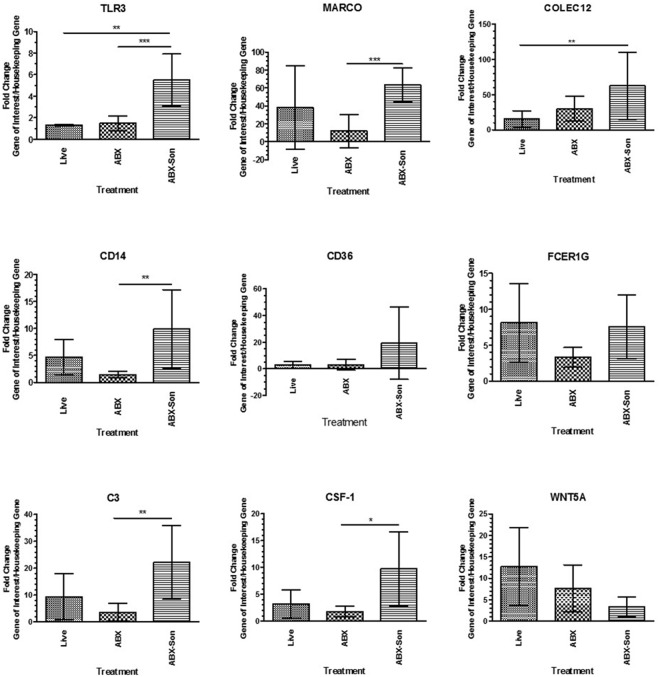
Validation of selected phagocytosis-related gene expression. Primary human microglia were stimulated with live virulent (V), non-virulent (NV), antibiotic-killed (Abx), or antibiotic-treated and sonicated (Abx/Son) *B. burgdorferi* for 24 h followed by RNA purification and cDNA synthesis. Individual primer sets (SABiosciences) were used to amplify transcripts of interest by qPCR. Data represent two biological replicates per time point, with each PCR reaction run in triplicate. Data were normalized to transcription levels of two independent housekeeping genes and are expressed as fold change compared to no spirochete control. Error bars represent SEM. Statistical significance for qPCR experiments was determined by running a one-way ANOVA with a *post hoc* Dunn’s test (^∗^*p* < 0.05, ^∗∗^*p* < 0.01, ^∗∗∗^*p* < 0.001).

We then validated the expression of selected differentially regulated genes by qPCR array analysis. The mRNA expression of selected genes from the phagocytosis array was analyzed at 24 h after *B. burgdorferi* stimulation. Transcriptional induction by antibiotic-killed and antibiotic-killed sonicated was observed by qRT-PCR and that seen in the commercial real-time PCR arrays for several transcripts, including MARCO and CD14 (**Figure 4**). In contrast to the array results, live *B. burgdorferi* also upregulated several of these transcripts, including CD14 and MARCO. No significant differences were seen between live and antibiotic-killed spirochete induction of transcripts, with the exception of TLR3.

### Confocal Microscopic Analysis of Microglial Phagocytosis of *B. burgdorferi*

**FIGURE 5:**
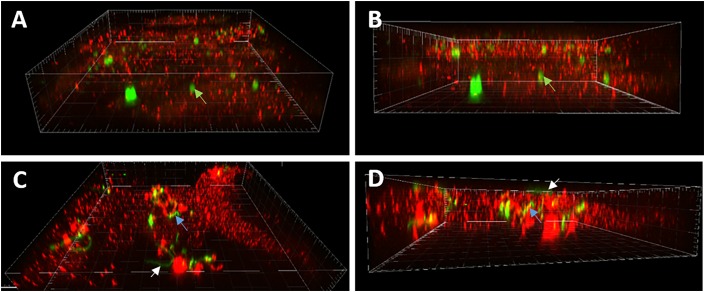
Confocal microscopy of *B. burgdorferi* interactions with primary human microglia. **(A)** Oblique view showing 1 μM green fluorescent beads associated with microglial cell (green arrows, 60X). **(B)** Demonstrates internalization of beads. Green arrows denote internalized bead in **(A,B)**. **(C)** White arrows illustrate intact spirochete associated with surface of microglial cell with no indication of internalization. **(D)** Blue arrows demonstrate internalization of *Borrelia* particles **(C,D)** suggesting internalization is associated with prior bacterial cell death.

In order to confirm whether the results of commercial phagocytosis arrays were the result of internalization or solely the aftermath of cell-surface receptor cascades, confocal microscopy was used to visualize *B. burgdorferi* in microglia cells. Primary cultures of human microglia were left un-stimulated, stimulated with green fluorescent control beads, or stimulated for 24 h with fluorescent *gfp*-expressing *B. burgdorferi.* After fixation, cells were stained with Texas Red 594 for IBA-1. Images of non-treated control cells demonstrate Texas Red 594 bound to IBA-1 without the co-localization of *gfp*-expressing *B. burgdorferi* (**Figure 5**). Fluorescent beads are useful probes for measuring phagocytic response among cells. Indeed, our cultures of primary human microglia are capable of internalizing or engulfing fluorescent control beads and confocal analysis of microglia treated with live *B. burgdorferi* suggested interaction on the cell surface in addition to internalization as well (**Figure 5**).

## Discussion

Previous work on murine microglia and *B. burgdorferi* indicates that both live *B. burgdorferi* as well as spirochetal antigen induces IL-6 and TNF-α secretion from these cells. Furthermore, *B. burgdorferi* antigen induces increased expression of several innate immune molecules and signaling factors such as NOD2, TLR2, and CD14 by murine microglia (Rasley et al., 2002; Sterka and Marriott, 2006; Sterka et al., 2006; Tauber et al., 2011). Likewise, after 24 h of incubation with live *B. burgdorferi*, primary rhesus microglia respond with increased transcription of chemokines and cytokines including IL-6 and IL-8. Among other products, primary rhesus microglia elaborate the proteins IL-6 and IL-8 (Myers et al., 2009; Ramesh et al., 2009). As with murine microglia, TLRs and their signaling pathways are involved in the response of rhesus microglia to *B. burgdorferi* (Bernardino et al., 2008; Parthasarathy and Philipp, 2013). Taken together, previous studies characterizing the response of both murine and primary rhesus microglia to *B. burgdorferi* indicate an upregulation of innate immune receptors such as toll like receptors along with the secretion of chemokines and cytokines including IL-6, IL-8, and TNF-α.

Our results demonstrate the robust transcription and protein secretion of both chemotaxic and potentially neuroinflammatory proteins by primary human microglial cells in response to *B. burgdorferi in vitro*, consistent with previous studies in mouse and monkey microglia (Rasley et al., 2002; Myers et al., 2009). CXCL-1 and IL-8 (potent neutrophil chemoattractants) were among the most upregulated, although secretion of CXCL-10 (T cell chemoattractant) and IL-6 were also observed. IL-6 can play both inflammatory and neurotrophic roles in CNS disorders (Suzuki et al., 2009), and whether it potentiates *B. burgdorferi*-induced damage or protects neurons in Lyme neuroborreliosis is unknown. The strongest protein response was observed in antibiotic-killed and sonicated spirochete treated cultures indicating that elevated protein expression can occur in response to dead or viable *B. burgdorferi*. These data suggest even after antibiotic killing, *B. burgdorferi* debris and dead bacteria could potentiate an inflammatory response in the absence of live organisms. This concept is supported by work by Parthasarathy et al. (2013) demonstrating inflammatory responses to non-viable *B. burgdorferi* in human oligodendrocytes, as well as studies in which inflammation remained after antibiotic treatment of *B. burgdorferi*-infected rhesus macaques (Parthasarathy et al., 2013; Crossland et al., 2017; Embers et al., 2017).

We also saw increased transcription and protein secretion of growth factors and neuroprotective factors including LIF. LIF is a neurotrophic cytokine that shares a receptor subunit with IL-6 and ciliary neurotrophic factor (Suzuki et al., 2009), and was recently shown to be required to prevent the propagation of secondary neurodegeneration after brain injury (Goodus et al., 2016). Microglial activation can be beneficial or detrimental, depending on the balance of pro-inflammatory factors, anti-inflammatory molecules, neurotrophins, and the magnitude of response. Factors involved in promoting glial inflammation in response to infection and injury include more than the usual suspects (e.g., inflammatory cytokines). Indeed, recent work supports roles for the neuropeptide substance P in inflammatory CNS disorders, including infection with *B. burgdorferi* (Chauhan et al., 2008; Johnson et al., 2016; Burmeister et al., 2017; Martinez et al., 2017). In light of recent work on the connection of glia to persistent pain (Loggia et al., 2015; Ji et al., 2016), the contribution of neurotrophins, growth factors, and neurotransmitters to cellular responses to *B. burgdorferi* may offer clues to chronic pain often reported by patients with post-treatment Lyme sequelae. Interestingly, we saw upregulation of CSF-1 in response to *B. burgdorferi*, and this factor has been recently linked to microglia proliferation (Guan et al., 2016).

We also investigated whether primary human microglia could effectively phagocytize *B. burgdorferi*. Phagocytosis of *B. burgdorferi* can occur via one of three mechanisms: opsonic mediated phagocytosis (through interaction with complement receptors), conventional phagocytosis mediated by integrins and C-type lectins, or coiling phagocytosis, which involves filopodial protrusion and actin rearrangement (reviewed in Cervantes et al., 2014). Many of the factors involved in phagocytosis of *B. burgdorferi* were substantially upregulated in primary human microglia, such as CD14 and MARCO, but only in the presence of dead spirochetes. These results were somewhat surprising, as murine microglia incubated with live *B. burgdorferi* can efficiently phagocytize and kill the bacteria, even in the absence of opsonizing antibodies (Kuhlow et al., 2005). The cell surface component CD14 is key to the recognition of bacteria such as *B. burgdorferi*. More specifically, CD14 has been shown to cooperate with complement receptor 3 in promoting phagocytosis of *B. burgdorferi* in both murine macrophages and human monocytes (Hawley et al., 2012). The scavenger receptor MARCO is upregulated in response to *B. burgdorferi* in murine macrophages, and the lack of this receptor decreases the ability of these cells to internalize *B. burgdorferi* (Petnicki-Ocwieja et al., 2013). In the only other published report investigating responses of human microglia to *B. burgdorferi*, cells stimulated with *B. burgdorferi* lysate also upregulate transcription of MARCO (Cassiani-Ingoni et al., 2006).

Consistent with these results, we saw increased expression of MARCO only in antibiotic-killed and antibiotic-killed sonicated *B. burgdorferi*-treated cells. MARCO and other scavenger receptors may play a role in Alzheimer’s and other neurodegenerative diseases, where the receptors facilitate uptake of amyloid beta and induce an inflammatory response that could contribute to microglial neurotoxicity (Yu and Ye, 2015). Future experiments are required to confirm the expression of MARCO in microglia in the rhesus macaque model of neuroborreliosis, and its function in *B. burgdorferi* clearance in non-murine models.

## Conclusion

We have demonstrated that primary human microglia treated with *B. burgdorferi* experience increased expression of pattern recognition receptors and genes known to be involved with cytoskeletal rearrangement and phagocytosis, in addition to the expected increased expression as well as secretion of pro-inflammatory molecules and neurotrophic factors. Our data also indicate that *B. burgdorferi* interacts with the cell surface of primary human microglia and may be internalized following this initial interaction. Our futures studies will use scanning and transmission electron microscopy to gather high-resolution images of the cell surface interacting with *B. burgdorferi* to confirm *B. burgdorferi* internalization by human microglia.

## Author Contributions

JG performed the experiments and this work represents a portion of his UND Undergraduate Honors Thesis. RG provided training for JG and assisted in the execution of the experiments. CB contributed to the training of JG and RG, as well as the design and conception of the experiments, statistical analysis, and preparation of the manuscript. JW also contributed to the training of JG and the conception, design, and execution of the experiments and preparation of the manuscript.

## Conflict of Interest Statement

The authors declare that the research was conducted in the absence of any commercial or financial relationships that could be construed as a potential conflict of interest.

## Abbreviations

CCL-5: chemokine C-C motif ligand
CD-14: cell differentiation factor 14
CNS: central nervous system
CSF: cerebral spinal fluid
CSF-1: macrophage colony stimulating factor-1
CXCL: chemokine C-X-C motif
ELISA: enzyme linked immunosorbent assay
GDF-5: growth differentiation factor-5
gfp: green fluorescent protein
IL-8: interleukin 8
LIF: leukemia inhibitory factor
MARCO: macrophage receptor with collagenous structure
MOI: multiplicity of infection
PBS: phosphate buffered saline
PCR: polymerase chain reaction
PLA: phospholipase
PTLDS: post-treatment Lyme disease syndrome
SCARB1: human scavenger receptor class B
TLR: toll-like receptor
VEGF: vascular epithelial growth factor
